# mRNA Delivery Platform Based on Bacterial Outer Membrane Vesicles for Tumor Vaccine

**DOI:** 10.21769/BioProtoc.4774

**Published:** 2023-07-05

**Authors:** Xiaoyu Gao, Yao Li, Guangjun Nie, Xiao Zhao

**Affiliations:** 1 CAS Key Laboratory for Biomedical Effects of Nanomaterials and Nanosafety & CAS Center for Excellence in Nanoscience, National Center for Nanoscience and Technology of China, 11 Beiyitiao, Zhongguancun, Beijing, 100190, China; 2 University of Chinese Academy of Sciences, Beijing, 100049, China; 3 Institute of Smart Biomedical Materials, School of Materials Science and Engineering, Zhejiang Sci-Tech University, Hangzhou 310018, China; 4 IGDB-NCNST Joint Research Center, Institute of Genetics and Developmental Biology, Chinese Academy of Sciences, Beijing, 100101, China

**Keywords:** RNA binding protein, BoxC/D, Cancer immunotherapy, mRNA vaccines, Outer membrane vesicles, Rapid display

## Abstract

The rapid display and delivery method for customized tumor mRNA vaccines is limited. Herein, bacteria-derived outer membrane vesicles (OMVs) are employed as an mRNA delivery platform by surface engineering of an RNA-binding protein, L7Ae. OMV-L7Ae can rapidly adsorb boxC/D sequence-labeled mRNA antigens through L7Ae-boxC/D binding and deliver them into HEK-293T and dendritic cells. This platform provides an mRNA delivery technology distinct from lipid nanoparticles (LNPs) for personalized mRNA tumor vaccination and with a *Plug-and-Display* strategy suitable for rapid preparation of the personalized mRNA tumor vaccine against varied tumor antigens.

Key features

OMVs are employed as an mRNA delivery platform through L7Ae-boxC/D binding.

**Graphical overview**


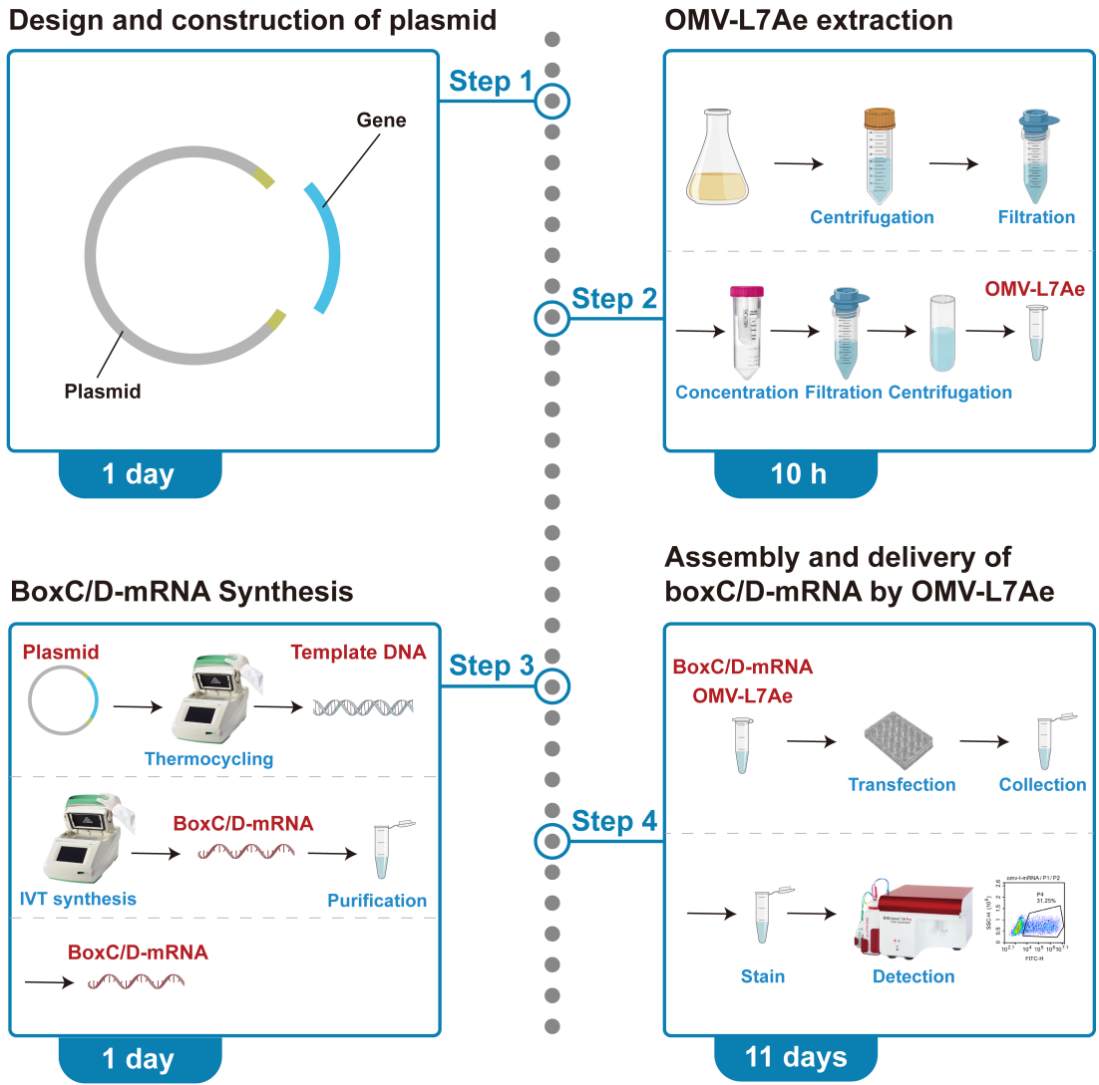


## Background

In recent years, mRNA vaccines have emerged as a promising weapon in cancer immunotherapy (G. Liu et al., 2021). Through precise sequence design, tumor mRNA vaccines encode one or more tumor-specific antigens derived from gene mutations in tumor cells. After vaccine uptake and intracellular protein translation in antigen presenting cells (APCs), tumor-specific antigens form complexes with the major histocompatibility complex I (MHCI) that are presented to T cells to activate a robust antitumor immunity (Miao et al., 2021). However, because of its poor stability, large molecular weight, and high negative charge, mRNA vaccines must rely on efficient delivery carriers to enter APCs (Miao et al., 2019; Barbieri and Kouzarides, 2020). So far, the mainstream mRNA carriers in the clinic are lipid nanoparticles (LNPs) (Liang and Zhao, 2021), which encapsulate mRNA in nanocarriers through a microfluid-based synthesis process (C. Liu et al., 2019; Blass and Ott, 2021; Dolgin, 2021). However, due to the heterogeneity and complexity of tumor antigens, the time-consuming encapsulation process is not suitable to produce personalized tumor vaccines (Wang et al., 2022).

Outer membrane vesicles (OMVs), as biogenic nanocarriers secreted by bacteria, are rich in bacterial components, with the ability to integrate adjuvants and carriers (Cheng et al., 2021; Liang et al., 2022; Yue et al., 2022; Zhao et al., 2022). In addition, OMVs can be obtained by bacterial fermentation in large quantities, which has attracted more and more attention (Cheng et al., 2020; Gao et al., 2022). Here, we describe a *Plug-and-Display* strategy for mRNA antigen delivery in an OMV-based platform by arming OMVs with an RNA-binding protein, L7Ae (Li et al., 2022). BoxC/D, the matched binding sequence, is added to the 3′-untranslated region (UTR) of the in vitro–transcribed (IVT) mRNA. The archaeal RNA-binding protein L7Ae is fused to the C-terminal of the surface protein ClyA on the OMVs (OMV-L7Ae). The boxC/D sequence adopts the standard k-turn conformation that is specifically recognized by the L7Ae protein, which stabilizes the stem-loop and forms a standard L7Ae-k-turn complex (Moore et al., 2004; Turner et al., 2005; Saito et al., 2010). Through the strong and specific binding between the L7Ae protein and the boxC/D sequence, the boxC/D-labeled mRNA (boxC/D-mRNA) is rapidly adsorbed onto the surface of the OMV-L7Ae, achieving the successful mRNA delivery into cells via endocytosis of OMVs by HEK-293T or bone marrow dendritic cells (BMDCs). This Plug-and-Display strategy is suitable for the rapid preparation of tumor mRNA vaccines against heterogeneous and complex tumor antigens.

## Materials and reagents

**Biological materials**

1. *E. coli* [strain BL21 (DE3)] (Tiangeng, catalog number: DHGST-21)
2. HEK-293T cells (American Type Culture Collection, catalog number: CRL-3216)
3. C57BL/6 mice (Vital River Laboratory Animal Technology, catalog number: 01059)

**Reagents**

1. NaCl (Solarbio, catalog number: S8210)
2. Yeast extract (Solarbio, catalog number: Y8020)
3. Tryptone (Solarbio, catalog number: T8490)
4. Agar (Solarbio, catalog number: A8190)
5. Chloramphenicol (50 mg/mL) (Solarbio, catalog number: L1311)
6. Isopropyl-β-d-thiogalactoside (IPTG) (Solarbio, catalog number: BS119)
*Note: IPTG causes serious eye irritation. Wear nitrile gloves, safety goggles, and lab coats, and operate carefully in a fume hood.*
7. Fetal bovine serum (FBS) (Wisent, catalog number: 085-150)
8. Penicillin G and streptomycin (Wisent, catalog number: 450-201-EL)
9. Phosphate-buffered saline (PBS) (Wisent, catalog number: 311-010-CL)
10. Dulbecco’s modified Eagle medium (DMEM) (Wisent, catalog number: 319-005-CL)
11. RPMI 1640 medium (Wisent, catalog number: 350-000-CL)
12. HiScribe T7 ARCA mRNA kit (with tailing) (New England Biolabs, catalog number: E2060S)
13. Q5 high-fidelity 2× master mix (New England Biolabs, catalog number: M0492S)
14. Tris-HCl (1 M, pH 8.0) (Solarbio, catalog number: T1150)
15. MgCl_2_ (1 M) (Beyotime, catalog number: ST269)
16. Diethylpyrocarbonate (DEPC)-treated water (Biosharp, BL510B)
17. Citric acid sodium citrate buffer (0.1 M, pH = 4.0) (Leagene, R00521)
18. Ethanol (Macklin, catalog number: E809063)
19. Ammonium chloride potassium (ACK) lysis buffer (Solarbio, catalog number: R1010)
20. HEPES (1 M, pH 7.4) (Sigma-Aldrich, catalog number: 83264)
21. β-Mercaptoethanol (β-ME) (Gibco, catalog number: 21985023)
22. IL-4 (Sino Biological, catalog number: 51084-MNAE)
23. Granulocyte-macrophage colony-stimulating factor (GM-CSF) (Sino Biological, catalog number: 51048-M01H)
24. FITC-anti-mouse CD11c (BioLegend, catalog number: 117306)
25. APC-anti-mouse CD80 (BioLegend, catalog number: 104713)
26. PE/Cy7-anti-mouse CD86 (BioLegend, catalog number: 105014)
27. APC-anti-mouse H-2Kb bound to SIINFEKL (BioLegend, catalog number: 141605)
28. pST1374-NLS-flag-linker-Cas9 plasmid (Addgene, catalog number: 44758)
29. Trypsin-EDTA (0.25%) (Meilunbio, catalog number: MB4376)

**Solutions**

1. Solid LB medium (see Recipes)
2. Liquid LB medium (see Recipes)
3. Reaction buffer for mRNA binding (pH = 5.0) (see Recipes)

## Recipes

1. **Solid LB medium**
  **Table:** 

Reagent	Final concentration	Amount
Yeast extract	5 g/L	n/a
Tryptone	10 g/L	n/a
NaCl	10 g/L	n/a
Agar	15 g/L	n/a
H_2_O	n/a	100 mL
Total	n/a	100 mL
  Prepare the solid LB medium by dissolving 1.5 g of agar, 1 g of NaCl, 1 g of tryptone, and 0.5 g of yeast extract in 100 mL of deionized water. After sterilization for 20 min at 121 °C and 0.1 MPa, add 100 μL of 50 mg/mL chloramphenicol when the temperature reaches approximately 60 °C. Add 10 mL of sterilized LB medium to a dish (10 cm) after fully mixing. After solidification, seal the solid LB medium with sealing film. Store the solid LB medium for one month at 4 °C.
*Note: Add antibiotics only after the temperature drops to ~50–60 °C, because their activity is seriously affected by temperature.*
2. **Liquid LB medium**
  **Table:** 

Reagent	Final concentration	Amount
Yeast extract	5 g/L	n/a
Tryptone	10 g/L	n/a
NaCl	10 g/L	n/a
H_2_O	n/a	1 L
Total	n/a	1 L
  Prepare the liquid LB medium by dissolving 10 g of tryptone, 5 g of yeast extract, and 10 g of NaCl in 1 L of deionized water. After sterilization for 20 min at 121 °C and 0.1 MPa, store the liquid LB medium for one month at 4 °C.
3. **Reaction buffer for mRNA binding (pH = 5.0)**
  **Table:** 

Reagent	Final concentration	Amount
NaCl	100 × 10^-3^ M	n/a
Tris-HCl (1 M, pH 8.0)	5 × 10^-3^ M	200 μL
MgCl_2_ (1 M)	10 × 10^-3^ M	400 μL
Total	n/a	40 mL
  Prepare the reaction buffer for mRNA binding by dissolving 233.76 mg of NaCl in 30 mL of DEPC-treated water. Then, add 200 μL of 1 M Tris-HCl (pH = 8.0) and 400 μL of 1 M MgCl_2_ and fully mix. After adjusting pH to 5.0 with citric acid sodium citrate buffer, set the volume to 40 mL. Seal the reaction buffer for mRNA binding with sealing film and store the reaction buffer for mRNA binding for one month at 4 °C.
*Note: To prevent the introduction of RNA enzymes, water must be DEPC treated.*

**Laboratory supplies**

1. Conical flask (250 mL) (Heqi Glass, catalog number: B-000207)
2. Conical flask (500 mL) (Heqi Glass, catalog number: B-000209)
3. Sterile breathable sealing film (Bkmam, catalog number: B-FK14-50E)
4. Culture plate (10 cm) (Corning, catalog number: 430167)
5. Centrifuge tube (50 mL) (Corning, catalog number: 430829)
6. Centrifuge tube (15 mL) (Corning, catalog number: 430791)
7. Centrifuge tube (1.5 mL) (Kirgen, catalog number: KG2211)
8. PCR tube (0.2 mL) (Thermo Scientific, catalog number: AB0620)
9. 6-well plate (Corning, catalog number: 3516)
10. 24-well plate (Corning, catalog number: 3524)
11. Sealing film (Parafilm, catalog number: PM-996)
12. Injection syringe (Acmec, catalog number: AYA0553)
13. Filter (0.45 μm) (Merck Millipore, catalog number: SLHPR33RB)
14. Filter (0.22 μm) (Merck Millipore, catalog number: SLGPR33RB)
15. Cell strainer (70 μm) (BD Falcon, catalog number: 352350)
16. Ultrafiltration tube (100 kDa) (Merck Millipore, catalog number: UFC910096)
17. Ultracentrifugation tube (Beckman, catalog number: 355618)
18. Quartz cell (Heqi Glass, catalog number: B-037003)

## Equipment

1. Water bath (BluePard, model: HWS-24)
2. Bacteriological incubator (BluePard, model: THZ-98C)
3. Clean bench (for bacteria) (Beijing Dinglian Har Instrument Manufacture, model: DL-CJ-1NDII)
4. Clean bench (for cell) (Thermo Fisher Scientific, catalog number: 1384)
5. High-speed centrifuge (Thermo Fisher Scientific, model: Sorvall ST 8R)
6. Ultra-speed centrifuge (Beckman, model: OPTIMA XPN-100)
7. Flow cytometer (Agilent Technologies, catalog number: 462171219542)
8. Spectrophotometer (Asone, model: ASV-S3)
9. Cell incubator (Thermo Fisher Scientific, catalog number: BB150)
10. pH meter (Mettler Toledo, catalog number: S210FE20K)
11. Refrigerator (-80 °C) (Haier, model: HYCD-290)
12. Refrigerator (4 °C and -20 °C) (Haier, model: DE-25W262)
13. T100 thermal cycler (Bio-Rad, catalog number: 621BR55553)
14. NanoDrop (Implen, model: N50 Touch)
15. Automated cell counter (Invitrogen, model: Countess 3)
16. Autoclave (STIK Instrument Equipment, model: IMJ-54A)

## Software

1. Prism 9 (GraphPad)

## Procedure

- **Plasmid design and construction (timing: 1 day)**
  1. Design and construction of pACYC-ClyA-L7AE plasmid:
    The following sequences are synthesized, sequentially ligated, and inserted into the pACYCDuet-1 plasmid by NdeI (CATATG) and XhoI (CTCGAG) (Table 1). The product is named pACYC-ClyA-L7AE.
    **Table 1.** Design of pACYC-ClyA-L7AE plasmid

DNAs	Sequences
ClyA	ATGACTGAAATCGTTGCAGATAAAACGGTAGAAGTAGTTAAAAACGCAATCGAAACCGCAGATGGAGCATTAGATCTTTATAATAAATATCTCGATCAGGTCATCCCCTGGCAGACCTTTGATGAAACCATAAAAGAGTTAAGTCGCTTTAAACAGGAGTATTCACAGGCAGCCTCCGTTTTAGTCGGCGATATTAAAACCTTACTTATGGATAGCCAGGATAAGTATTTTGAAGCAACCCAAACAGTGTATGAATGGTGTGGTGTTGCGACGCAATTGCTCGCAGCGTATATTTTGCTATTTGATGAGTACAATGAGAAGAAAGCATCCGCCCAGAAAGACATTCTCATTAAGGTACTGGATGACGGCATCACGAAGCTGAATGAAGCGCAAAAATCCCTGCTGGTAAGCTCACAAAGTTTCAACACGCTTCCGGGAAACTGCTGGCGTTAGATAGCCAGTTAACCAATGATTTTTCAGAAAAAAGCAGCTATTTCCAGTCACAGGTAGATAAAATCAGGAAGGAAGCGTATGCCGGTGCCGCAGCCGGTGTCGTCGCCGGTCCATTTGGATTAATCATTTCCTATTCTATTGCTGCGGGCGTAGTTGAAGGAAAACTGATTCCAGAATTGAAGAACAAGTTAAAATCTGTGCAGAATTTCTTTACCACCCTGTCTAACACGGTTAAACAAGCGAATAAAGATATCGATGCCGCCAAATTGAAATTAACCACCGAAATAGCCGCCATCGGTGAGATAAAAACGGAAACTGAAACAACCAGATTCTACGTTGATTATGATGATTTAATGCTTTCTTTGCTAAAAGAAGCGGCCAAAAAAATGATTAACACCTGTAATGAGTATCAGAAAAGACACGGTAAAAAGACACTCTTTGAGGTACCTGAAGTC
Linker	GGTGGTGGATCA
L7Ae	ATGTACGTGAGATTTGAGGTTCCTGAGGACATGCAGAACGAAGCTCTGAGTCTGCTGGAGAAGGTTAGGGAGAGCGGTAAGGTAAAGAAAGGTACCAACGAGACGACAAAGGCTGTGGAGAGGGGACTGGCAAAGCTCGTTTACATCGCAGAGGATGTTGACCCGCCTGAGATCGTTGCTCATCTGCCCCTCCTCTGCGAGGAGAAGAATGTGCCGTACATTTACGTTAAAAGCAAGAACGACCTTGGAAGGGCTGTGGGCATTGAGGTGCCATGCGCTTCGGCAGCGATAATCAACGAGGGAGAGCTGAGAAAGGAGCTTGGAAGCCTTGTGGAGAAGATTAAAGGCCTTCAGAAG
Linker	GGTGGCGGATCA
3× HA tag	TACCCATACGATGTTCCAGATTACGCTTATCCCTACGACGTGCCTGATTATGCATACCCATACGATGTCCCCGACTATGCC
Termination codon	TAA
*Notes:*
    - *The pACYCDuet-1 plasmid is only a suggested plasmid, which can be replaced by other suitable plasmids.*
    - *The principle of plasmid selection: (1) choose a small-molecular-weight plasmid, so that the plasmid is more stable and with a higher copy number; (2) choose a relaxation control plasmid, so that the plasmid can replicate autonomously; (3) choose a plasmid with multiple restriction enzyme cut points; (4) choose a plasmid with easy-to-detect markers, such as resistance markers; and (5) choose a plasmid expressed in E. coli.*
  2. Design and construction of pST1374-EGFP-boxC/D plasmid:
    The following sequences are synthesized, sequentially ligated, and inserted into pST1374-NLS-flag-linker-Cas9 plasmid by NdeI (CATATG) and ApaI (GGGCCC) (Table 2). The product is named pST1374-EGFP-boxC/D.
    **Table 2.** Design of pST1374-EGFP-boxC/D plasmid

DNAs	Sequences
5′-UTR	GGGAAATAAGAGAGAAAAGAAGAGTAAGAAGAAATATAAGAGCCACC
EGFP	ATGGTGAGCAAGGGCGAGGAGCTGTTCACCGGGGTGGTGCCCATCCTGGTCGAGCTGGACGGCGACGTAAACGGCCACAAGTTCAGCGTGTCCGGCGAGGGCGAGGGCGATGCCACCTACGGCAAGCTGACCCTGAAGTTCATCTGCACCACCGGCAAGCTGCCCGTGCCCTGGCCCACCCTCGTGACCACCCTGACCTACGGCGTGCAGTGCTTCAGCCGCTACCCCGACCACATGAAGCAGCACGACTTCTTCAAGTCCGCCATGCCCGAAGGCTACGTCCAGGAGCGCACCATCTTCTTCAAGGACGACGGCAACTACAAGACCCGCGCCGAGGTGAAGTTCGAGGGCGACACCCTGGTGAACCGCATCGAGCTGAAGGGCATCGACTTCAAGGAGGACGGCAACATCCTGGGGCACAAGCTGGAGTACAACTACAACAGCCACAACGTCTATATCATGGCCGACAAGCAGAAGAACGGCATCAAGGTGAACTTCAAGATCCGCCACAACATCGAGGACGGCAGCGTGCAGCTCGCCGACCACTACCAGCAGAACACCCCCATCGGCGACGGCCCCGTGCTGCTGCCCGACAACCACTACCTGAGCACCCAGTCCGCCCTGAGCAAAGACCCCAACGAGAAGCGCGATCACATGGTCCTGCTGGAGTTCGTGACCGCCGCCGGGATCACTCTCGGCATGGACGAGCTGTACAAGTAA
3′-UTR	TGATAATAGGCTGGAGCCTCGGTGGCCATGCTTCTTGCCCCTTGGGCCTCCCCCCAGCCCCTCCTCCCCTTCCTGCACCCGTACCCCCGTGGTCTTTGAATAAAGTCTGA
BoxC/D	GGGCGTGATGCGAAAGCTGACCCGGGCGTGATGCGAAAGCTGACCCGCTCTGACCGAAAGGCGTGATGAGCGCTCTGACCGAAAGGCGTGATGAGC
*Notes:*
    - *The pST1374-NLS-flag-linker-Cas9 plasmid is only a suggested plasmid, which can be replaced by other suitable plasmids.*
    - *The principle of plasmid selection: (1) choose a small-molecular-weight plasmid, so that the plasmid is more stable and with a higher copy number; (2) choose a relaxation control plasmid, so that the plasmid can replicate autonomously; (3) choose a plasmid with multiple restriction enzyme cut points; (4) choose a plasmid with easy-to-detect markers, such as resistance markers; and (5) choose a plasmid expressed in E. coli.*
  3. Design and construction of pST1374-sec-OVA-3HA-MITD- boxC/D plasmid:
    The following sequences are synthesized, sequentially ligated, and inserted into pST1374-NLS-flag-linker-Cas9 plasmid by NdeI (CATATG) and ApaI (GGGCCC) (Table 3). The product is named pST1374-sec-OVA-3HA-MITD-boxC/D.
    **Table 3.** Design of pST1374-sec-OVA-3HA-MITD-boxC/D plasmid

DNAs	Sequences
5′-UTR	GGGAAATAAGAGAGAAAAGAAGAGTAAGAAGAAATATAAGAGCCACC
Sec	ATGGTACCGTGCACGCTGCTCCTGCTGTTGGCGGCCGCCCTGGCTCCGACTCAGACCCGCGCG
Linker	GGCGGTTCTGGAGGGGGTGGGTCCGGGGGT
OVA	CAGCTTGAGAGTATAATCAACTTTGAAAAACTGACT
3 HA	TACCCATACGATGTTCCAGATTACGCTTATCCCTACGACGTGCCTGATTATGCATACCCATATGATGTCCCCGACTATGCC
MITD	GCGACCGTTGCTGTTCTGGTTGTCCTTGGAGCTGCAATAGTCACTGGAGCTGTGGTGGCTTTTGTGATGAAGATGAGAAGGAGAAACACAGGTGGAAAAGGAGGGGACTATGCTCTGGCTCCAGGCTCCCAGACCTCTGATCTGTCTCTCCCAGATTGTAAAGTGATGGTTCATGACCCTCATTCTCTAGCGTGA
3′-UTR	GCTCGCTTTCTTGCTGTCCAATTTCTATTAAAGGTTCCTTTGTTCCCTAAGTCCAACTACTAAACTGGGGGATATTATGAAGGGCCTTGAGCATCTGGATTCTGCCTAATAAAAAACATTTATTTTCATTGCGCTCGCTTTCTTGCTGTCCAATTTCTATTAAAGGTTCCTTTGTTCCCTAAGTCCAACTACTAAACTGGGGGATATTATGAAGGGCCTTGAGCATCTGGATTCTGCCTAATAAAAAACATTTATTTTCATTGC
BoxC/D	GGGCGTGATGCGAAAGCTGACCCGGGCGTGATGCGAAAGCTGACCCGCTCTGACCGAAAGGCGTGATGAGCGCTCTGACCGAAAGGCGTGATGAGC
*Notes:*
    - *The pST1374-NLS-flag-linker-Cas9 plasmid is only a suggested plasmid, which can be replaced by other suitable plasmids.*
    - *The principle of plasmid selection: (1) choose a small-molecular-weight plasmid, so that the plasmid is more stable and with a higher copy number; (2) choose a relaxation control plasmid, so that the plasmid can replicate autonomously; (3) choose a plasmid with multiple restriction enzyme cut points; (4) choose a plasmid with easy-to-detect markers, such as resistance markers; and (5) choose a plasmid expressed in E. coli.*
  4. The above plasmids are synthesized by GENEWIZ, Suzhou, China (https://www.genewiz.com.cn/).
- **Plasmid transformation and bacterial culture (timing: 3 days)**
  1. Place a centrifuge tube (1.5 mL) containing 100 μL of competent cells [*E. coli* BL21 (DE3)] in an ice bath until completely thawed.
*Notes:*
    - *Store the competent cells [E. coli BL21 (DE3)] at -80 °C.*
    - *Avoid freezing and thawing to ensure the activity of the competent cells.*
    - *After thawing the competent cells, DNA should be quickly added.*
  2. Add 1 ng of pACYC-ClyA-L7AE plasmid into the competent cells. Mix gently and let stand in an ice bath for 30 min.
  3. Place the centrifuge tube for 60–90 s in a 42 °C water bath. Transfer the centrifuge tube quickly to an ice bath for 2–3 min to cool the cells.
*Note: Do not shake the centrifugal tube during this process.*
  4. Add 900 μL of liquid LB medium to the centrifuge tube. Incubate for 45–60 min at 37 °C in a shaker at 150 rpm.
*Notes:*
    - *The resistance gene has not yet been expressed during this process, so the liquid LB medium must be antibiotic-free.*
    - *Growth for 1 h at 37 °C has the best effect on cell recovery and antibiotic resistance expression.*
  5. Add 100 μL of transformed cells to a culture plate (10 cm) with solid LB medium containing 50 μg/mL chloramphenicol. Spread the cells gently with a sterile glass stick.
*Note: Prepare the culture plate (10 cm) with the solid LB medium containing 50 μg/mL chloramphenicol in advance.*
  6. Place the culture plate at room temperature until the liquid is absorbed. Invert the culture plate and culture for 12–16 h at 37 °C. The culture plate should be sealed with sterile sealing film (Troubleshooting 1).
*Note: The number of bacteria can be adjusted on the plate, ideally to obtain several dozens of colonies on a plate (10 cm).*
    **Pause point:** Store the bacteria on the plate for one month at 4 °C.
  7. Carefully pick one bacterial colony on the plate. Add it into 3 mL of liquid LB medium containing 50 μg/mL chloramphenicol in a centrifuge tube (15 mL). Incubate for 10–12 h at 37 °C with shaking at 180 rpm.
*Note: To ensure oxygen supply for bacterial growth, seal the centrifuge tube using the sterile breathable sealing film.*
  8. Add 3 mL of bacterial culture medium from step B7 into 300 mL of liquid LB medium containing 50 μg/mL chloramphenicol in a conical flask (500 mL). Incubate at 37 °C with shaking at 180 rpm. A spectrophotometer is used to monitor the optical density at 600 nm (OD600) of the bacterial culture medium. Add 0.1 mM IPTG to the medium when the OD600 reaches 0.6. Incubate for another 16 h at 16 °C with shaking at 180 rpm.
*Note: The IPTG-induced expression can be performed for 2 h at 37 °C, but the expression efficiency is not as good as induction at 16 °C.*
- **OMV-L7Ae extraction (timing: 10 h)**
  1. Divide the 300 mL bacterial medium into six centrifuge tubes (50 mL). After centrifugation at 7,000× *g* for 15 min at 4 °C, collect the supernatant.
*Notes:*
    - *Balance the weight of the centrifugal tubes (50 mL).*
    - *Dispose as soon as possible after centrifugation to prevent bacteria from spreading into the supernatant.*
  2. Filter the 300 mL supernatant through a filter (0.45 μm). Through centrifugation at 3,000× *g* for 5–10 min at 4 °C, concentrate the filtering medium to 100 mL using an ultrafiltration tube (100 kDa).
  3. Filter the concentrated solution through a filter (0.22 μm).
  4. Put the filtering medium into two ultracentrifugation tubes.
*Note: Clean and dry the ultracentrifugation tubes in advance.*
  5. Separate the OMV-L7Ae through ultracentrifugation at 150,000× *g* for 3 h at 4 °C.
*Notes:*
    - *Balance the weight of the ultracentrifugation tubes; the weight error of all ultracentrifugation tubes is less than 50 mg.*
    - *Tighten the ultracentrifugation tube cover.*
  6. Discard the supernatant. To fill up the ultracentrifugation tubes, resuspend the deposited OMV-L7Ae at the bottom of the tubes using DEPC-treated PBS. Repeat the ultracentrifugation at 150,000× *g* for 3 h at 4 °C.
  7. Discard the supernatant. Resuspend the deposited OMV-L7Ae with the 200 μL of reaction buffer for mRNA binding in each tube (Troubleshooting 2).
*Note: After ultracentrifugation, discard the supernatant as soon as possible to prevent the OMV-L7Ae from redissolving in the supernatant.*
    **Pause point:** Store the OMV-L7Ae for one week at -80 °C.
- **BoxC/D-mRNA synthesis (timing: 1 day)**
*Notes:*
  - *The following method is a reference to protocols from NEB’s official website (https://www.neb.com/).*
  - *Before the experiment, the table should be wiped with DEPC-water to avoid RNase contamination. Please wear gloves during the entire process. Be sure to use nuclease-free tubes and reagents to avoid RNase contamination.*
    1. Preparation of template DNA:
      - Design of primers (Table 4).
        **Table 4.** Design of primers

DNAs	Sequences
Forward primer	CTGGCTAACTAGAGAACCCAC
Reverse primer	CTAGAAGGCACAGTCGAGGCTG
      - Put the following PCR reaction master mix into a PCR tube (0.2 mL) (Table 5).
        **Table 5.** PCR reaction master mix

Reagent	Amount	Final Concentration
Q5 high-fidelity 2× master mix	25 μL	1×
10 μM forward primer	2.5 μL	0.5 μM
10 μM reverse primer	2.5 μL	0.5 μM
Plasmid (pST1374-EGFP-boxC/D)/plasmid (pST1374-sec-OVA-3HA-MITD-boxC/D)	variable	0.4 ng/μL
Nuclease-free water	to 50 μL	/
*Notes:*
*i. The Q5 high-fidelity 2× master mix should be thawed on ice to prevent inactivation.*
*ii. All the reaction components are operated on ice, and all components should be mixed prior to use.*
      - Collect all liquid to the tube bottom by a quick spin and quickly transfer the reactions to a thermal cycler preheated to the denaturation temperature (98 °C).
      - Begin PCR thermocycling (Table 6).
        **Table 6.** PCR cycling conditions

Steps	Temperature	Time	Cycles
Initial denaturation	98 °C	30 s	1
Denaturation	98 °C	7 s	35 cycles
Annealing	60 °C	20 s	
Extension	72 °C	25 s	
Final extension	72 °C	2 min	1
Hold	4 °C	Forever
      - Determine the concentration of PCR product using NanoDrop. Generally, the concentration is greater than 600 ng/μL (Troubleshooting 3).
        **Pause point:** Store the PCR product for one month at -20 or -80 °C.
    2. IVT synthesis of boxC/D-mRNA:
      - Prepare IVT mix into a PCR tube (0.2 mL) (Table 7).
        **Table 7.** IVT mix

Reagent	Amount	Final Concentration
Template DNA (PCR product from step D1)	variable	0.05 μg/μL
2× ARCA/NTP mix	10 μL	1×
T7 RNA polymerase mix	2 μL	/
Nuclease-free water	to 20 μL	/
*Notes:*
*i. All components should be thawed on ice to prevent inactivation.*
*ii. All the reaction components are operated on ice, and all components should be mixed prior to use.*
      - Collect all liquid to the tube bottom with a quick spin. Incubate for 30 min at 37 °C.
*Note: A longer reaction time would help to produce more IVT product.*
        **Pause point:** After the IVT reaction, store the product for one week at -20 °C.
      - Add 2 μL of DNase I to the IVT product, mix well, and incubate for 15 min at 37 °C to remove template DNA.
      - Prepare the following poly(A) tailing mix into a PCR tube (0.2 mL) (Table 8).
        **Table 8.** Poly(A) tailing mix

Reagent	Amount
IVT product from step D2c	20 μL
10× Poly(A) polymerase reaction buffer	10 μL
Poly(A) polymerase	5 μL
Nuclease-free water	to 100 μL

*Note: The unpurified IVT product contains enough ATP; no extra ATP is necessary for the poly(A) tailing reaction.*
      - Collect all liquid to the tube bottom with a quick spin. Incubate for 30 min at 37 °C.
    3. BoxC/D-mRNA purification:
      - To the 50 μL poly(A) tailing product, add 25 μL of LiCl solution and mix well.
      - Incubate for 30 min at -20 °C.
      - Centrifuge at 20,000× *g* for 15 min at 4 °C to pellet the boxC/D-mRNA.
      - Remove the supernatant carefully. Rinse the pellet by adding 500 μL of cold 70% ethanol.
*Note: 70% ethanol should be pre-cooled to -20 °C in advance.*
      - Centrifuge at 20,000× *g* for 10 min at 4 °C.
      - Remove the ethanol carefully. Spin the tube briefly to bring down any liquid on the wall. Remove residual liquid carefully using a sharp tip (e.g., loading tip).
*Note: The residual liquid should be removed to prevent organic solvents from affecting subsequent experiments.*
      - Air dry the pellet and resuspend the boxC/D-mRNA in 50 μL of DEPC-treated water.
      - Heat the boxC/D-mRNA for 5 min at 65 °C to completely dissolve the boxC/D-mRNA. Mix well.
      - Determine the boxC/D-mRNA concentration using NanoDrop. Generally, the boxC/D-mRNA concentration is approximately 500 ng/μL. The mRNA from plasmid pST1374-EGFP-boxC/D is named EGFP-boxC/D mRNA and the mRNA from plasmid pST1374-sec-OVA-3HA-MITD-boxC/D is named OVA-boxC/D mRNA (Troubleshooting 4).
*Note: The 260/280 of mRNA is between 1.8 and 2.0; 260/230 ≥ 2 indicates that the prepared mRNA is qualified.*
        **Pause point:** Store the boxC/D-mRNA for one month at -20 or -80 °C.
- **Assembly and delivery of boxC/D-mRNA by OMV-L7Ae (timing: 11 days)**
  1. Assembly of boxC/D-mRNA and OMV-L7Ae:
    Mix 9 μg of OMV-L7Ae (total protein weight) from step C7 and 1 μg of boxC/D-mRNA (EGFP-boxC/D or OVA-boxC/D) from step D3i and then put the mixture (OMV-L-mRNA) for 5 min at room temperature.
*Notes:*
    - *OMV-L7Ae and boxC/D-mRNA should be mixed immediately before delivery.*
    - *OMV-L7Ae vesicles should be free of bacterial contamination.*
  2. Delivery and evaluation of EGFP-boxC/D mRNA by OMV-L7Ae:
    - Before transfection experiments of 18–24 h, add HEK-293T cells to a 24-well plate at a density of 40,000 cells per well with 500 μL of DMEM medium containing 10% FBS, 100 U/mL penicillin G, and 100 μg/mL streptomycin. The cells are grown at 37 °C in the cell incubator with 5% CO_2_.
    - Supplement the OMV-L-mRNA mixture (EGFP-boxC/D mRNA) from step E1 with 500 μL of DMEM medium.
    - Carefully discard the cell supernatant of the 24-well plate.
*Note: The cell density is approximately 80% when transfection experiments are performed.*
    - Add 500 μL of OMV-L-mRNA mixture from step E2b to the HEK-293T cells in the 24-well plate.
    - Culture cells for 6–8 h at 37 °C in a humidified atmosphere with 5% CO_2_.
    - Carefully discard the cell supernatant of 24-well plate. Add 500 μL of DMEM medium containing 10% FBS, 100 U/mL penicillin G, and 100 μg/mL streptomycin to the 24-well plate.
    - At 24 h after transfection, digest the cells with 100 μL of trypsin-EDTA (0.25%) for 30 s. Add 300 μL of RPMI 1640 medium containing 10% FBS, 100 U/mL penicillin G, and 100 μg/mL streptomycin to stop digestion.
    - Through centrifugation at 800× *g* for 5 min at 4 °C, collect the HEK-293T cells.
    - Discard the supernatant and then resuspend the HEK-293T cells with 200 μL of PBS. Perform the flow cytometry evaluation within 1 h and analyze the cells expressing EGFP (Troubleshooting 5).
*Note: HEK-293T cells are fragile and should be handled gently.*
  3. Immune stimulation evaluation of OMV-based nanovaccines in vitro through delivery of OVA-boxC/D mRNA by OMV-L7Ae:
    - Keep the C57BL/6 mice (6–8 weeks old) with a 12 h light/dark cycle and a humidity of 30%–70% at 20–22 °C in a room. Provide food and water ad libitum.
    - After killing the C57BL/6 mice by cervical dislocation, dissect the mice and obtain the femurs and tibias. Use scissors and tweezers to remove as much muscle tissue as possible around the bones.
*Notes:*
*i. The femur has more bone marrow than the tibia.*
*ii. Do not destroy the bones to avoid pollution.*
    - Soak the bones in a sterile culture plate containing 70% alcohol for 3 min to disinfect and sterilize and then wash twice with sterile PBS.
    - Flush repeatedly the bone marrow cells with an injection syringe containing RPMI 1640 medium, 100 U/mL penicillin G, 100 μg/mL streptomycin, and 2% FBS until the bones are completely white.
*Note: The femurs and tibias of one mouse are cultured using approximately 40 mL of medium.*
    - Through centrifugation at 800× *g* for 5 min at 4 °C, collect the bone marrow cells.
    - Discard the supernatant. To lysis the red blood cells, resuspend the cells with 1 mL of ACK lysis buffer. Incubate for 90 s at room temperature.
    - Stop the lysis. Add 3 mL of PBS and then filter the cells through a cell strainer (70 μm).
    - Through centrifugation at 800× *g* for 5 min at 4 , collect the cells.
    - Discard the supernatant. Resuspend the precipitated cells with 12 mL of RPMI 1640 medium supplemented with 10% FBS, 100 U/mL penicillin G, 100 μg/mL streptomycin, 1% HEPES, 0.05 mM β-ME, 20 ng/mL IL-4, and 20 ng/mL GM-CSF, and then divide into six wells in a 6-well plate.
*Notes:*
*i. During steps E3a–E3i, make sure all reagents and samples are placed on an ice bath, as this has a positive effect on cell activity.*
*ii. To maintain a sterile state, perform all steps on a clean bench using sterile containers.*
    - Culture the cells for six days at 37 °C in the cell incubator with 5% CO_2_.
*Notes:*
*i. Replace half of the medium every 2–3 days.*
*ii. Remove and add medium gently to avoid interfering with cell growth.*
    - Collect non-adherent cells on day 6 through centrifugation at 800× *g* for 5 min at 4 , which are known as BMDCs.
    - Discard the supernatant and then resuspend the precipitated BMDCs with 10 mL of RPMI 1640 medium.
    - Quantify the cell density using the automated cell counter. Place 100,000 BMDCs into a centrifuge tube (1.5 mL) with 500 μL of RPMI 1640 medium.
    - Add OMV-L-mRNA mixture (OVA-boxC/D mRNA) from step E1 to the BMDCs in the centrifuge tube (1.5 mL).
    - Culture cells for 6–8 h at 37 °C in a humidified atmosphere with 5% CO_2_.
    - Centrifuge at 800× *g* for 5 min at 4 °C.
    - Carefully discard the cell supernatant. Add 500 μL of RPMI 1640 medium containing 10% FBS, 100 U/mL penicillin G, and 100 μg/mL streptomycin to the centrifuge tube (1.5 mL).
    - At 12 or 24 h after transfection, collect the stimulated BMDCs through centrifugation at 800× *g* for 5 min at 4 .
*Note: Seal the centrifuge tube (1.5 mL) with sealing film but preserve the air hole to ensure oxygen supply.*
    - Discard the supernatant and then resuspend the precipitated BMDCs with 200 μL of RPMI 1640 medium containing 2% FBS.
    - Add the proper antibodies to analyze the maturation and cross-presentation of BMDCs. For the maturation assay, stain the BMDCs with FITC-anti-mouse CD11c (1:200, 1 μL), APC-anti-mouse CD80 (1:200, 1 μL), or PE/Cy7-anti-mouse CD86 (1:200, 1 μL). For the cross-presentation assay, stain the BMDCs with FITC-anti-mouse CD11c (1:200, 1 μL) and APC-anti-mouse H-2Kb bound to SIINFEKL (MHCI-OVA) (1:80, 2.5 μL).
    - Stain the BMDCs under dark conditions for 30 min at 4 °C and then add 500 μL of RPMI 1640 medium containing 2% FBS, 100 U/mL penicillin G, and 100 μg/mL streptomycin to stop staining and wash cells.
    - Through centrifugation at 800× *g* for 5 min at 4 , collect the stained BMDCs. Discard the supernatant and then resuspend cells with 200 μL of PBS.
    - Perform the flow cytometry evaluation according to the manufacturer’s protocols within 1 h.
*Note: Using the CD80 or CD86 as the maturation marker, the CD80^+^ or CD86^+^ cells are gated in the CD11c^+^ cells. For the cross-presentation assay, the CD11c^+^MHCI-OVA^+^ cells are gated in the BMDCs.*

**Expected outcomes**

**Figure 1.**
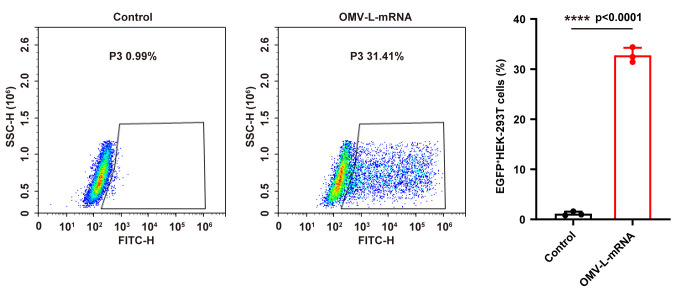
Flow cytometry analysis of HEK-293T cells incubated with PBS or outer membrane vesicle (OMV)-L-mRNA (2 μg/mL EGFP-boxC/D mRNA) for 24 h (n = 3). Data are shown as mean ± SD. One-way ANOVA and a Tukey’s multiple comparisons test were used for statistical analysis. ****, p < 0.0001. Figure adapted with permission from Li et al. (2022).

The flow cytometry results show that the OMV-L-mRNA (EGFP-boxC/D) successfully delivered the EGFP-boxC/D mRNA into HEK-293T cells, resulting in EGFP expression in 29.4% of cells (Figure 1, Figure S1).

**Figure 2.**
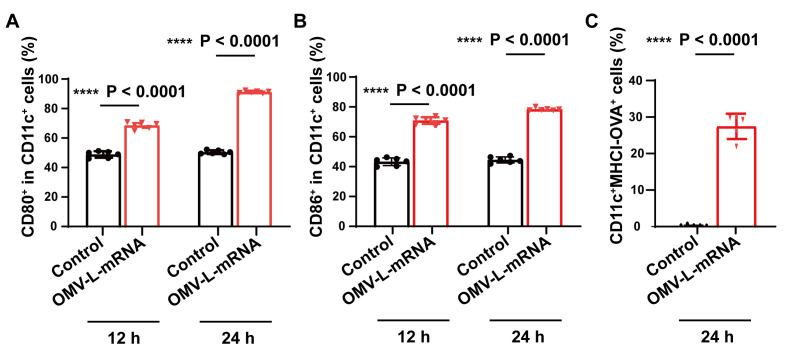
Innate immunity activation and antigen presentation mediated by the outer membrane vesicles (OMV)-based mRNA nanovaccines in vitro. (A, B) Bone marrow dendritic cells (BMDCs) maturation induced by PBS or OMV-L-mRNA (2 μg/mL OVA-boxC/D mRNA) for 12 or 24 h. The expression of CD80 (A) or CD86 (B) in CD11c^+^ BMDCs was examined using flow cytometry (n = 6). (C) Expression of the MHCI-OVA complex in BMDCs, as measured using flow cytometry (n = 5). The BMDCs were treated with PBS or OMV-L-mRNA (2 μg/mL OVA-boxC/D mRNA) for 24 h. The CD11c^+^MHCI-OVA^+^ cells in BMDCs were examined using flow cytometry. Data are shown as mean ± SD. One-way ANOVA and a Tukey’s multiple comparisons test were used for statistical analysis. ****, p < 0.0001. Figure adapted with permission from Li et al. (2022).

In the immune stimulation evaluation in BMDCs in step E3, the OMV-L-mRNA (OVA-boxC/D) induced notable maturation and antigen presentation, indicated by the upregulation of the surface expression of CD80, CD86, and MHCI-OVA (Figure 2A–2C, Figures S2–S3). These data demonstrated that the OMV-based nanocarriers can efficiently deliver mRNA antigens into BMDCs and induce antigen presentation.

**Limitations**

This study preliminarily demonstrated the feasibility and effectiveness of OMVs as tumor vaccine carriers for mRNA delivery. However, compared with the LNPs-based delivery platform, the efficiency of the OMV-based delivery platform is lower, which needs to be solved urgently in the subsequent clinical transformation process. We can make efforts in the following two aspects: 1) optimize the structure of the archaeal RNA-binding protein L7Ae and the matched binding sequence boxC/D, and 2) more boxC/D sequences can be connected in series to ensure greater binding and delivery efficiency.

In addition, as the bacteria-derived nanobiomaterials, the sterile production of the OMVs requires special attention in future clinical applications. In the procedure for the OMV-based nanocarriers, the bacteria were first centrifuged at 7,000× *g* for 15 min and almost all the bacteria were precipitated and removed. The OMVs from the supernatant were then filtered twice in 0.45 and 0.22 μm filters to ensure that the final OMVs did not contain bacteria. However, in future clinical applications, radiation sterilization of the final nanocarriers system can be performed to further ensure that the OMV nanocarriers are sterile.

## General notes and troubleshooting

**Troubleshooting**

**Problem 1**

There are too many or very few bacterial colonies on the plate.

**Potential solution**

1. This is one of the most common reasons for failure of the protocol. In many cases, the plasmid resistance genes were not associated with antibiotic, so the plasmid could not be transformed. We need to choose the right antibiotic based on the plasmid resistance genes in the backbone plasmid.
2. During competent cell resuscitation, the resistance genes were not expressed, and the LB medium with antibiotics was used (step B4), resulting in the failure of plasmid transformation. Therefore, the LB medium must be antibiotic-free in this step.
3. In the reagent setup of solid LB medium, the appropriate concentration of antibiotics was selected. If the concentration of antibiotics is too high, the target cells will not grow; if the concentration of antibiotics is too low, the growth of other bacteria will not be inhibited, resulting in the failure of plasmid transformation. In step B5, the volume of cells can be adjusted. When the volume of cells is too high, the cells will be too dense on the plate; when the volume of cells is too low, the target cells will not grow, resulting in the failure of plasmid transformation.

**Problem 2**

The amount of extracted OMV-L7Ae is very low.

**Potential solution**

1. Pick the single bacterial colony on the antibiotic plate as the culture source, not from the cryopreserved bacteria in glycerin.
2. After ultracentrifugation, the OMV-L7Ae was not treated as quickly as possible, resulting in redissolution of the OMV-L7Ae in the supernatant.

**Problem 3**

The template DNA preparation was not successful.

**Potential solution**

Q5 high-fidelity 2× master mix is highly susceptible to inactivation by repeated freezing and thawing. On the first use, the Q5 high-fidelity 2× master mix was partitioned. Alternatively, replace the Q5 high-fidelity 2× master mix.

**Problem 4**

The production of boxC/D-mRNA is low in the transcription reaction.

**Potential solution**

Determine the correct concentration of PCR product from step D1. In the preparation, nuclease-free water was used to reduce the introduction of RNase.

**Problem 5**

The transfection of HEK-293T cells was unsuccessful.

**Potential solution**

1. HEK-293T cells are fragile and should be handled gently in transfection experiments. The density of HEK-293T cells reached 80% when transfected.
2. OMV-L7Ae and boxC/D-mRNA (EGFP-boxC/D) may be placed for too long, resulting in mRNA degradation. OMV-L7Ae and boxC/D-mRNA (EGFP-boxC/D) can be extracted again to ensure that boxC/D-mRNA (EGFP-boxC/D) is not degraded.
3. The solution and equipment used in the experiment need to be fully rinsed with DEPC-treated water; ensure that there is no bacterial contamination.

## Supplementary information

The following supporting information can be downloaded here:

1. Figures S1: Gating strategy and representative scatter plots for analysis of transfection in HEK-293T cells (cf. Figure 1)
2. Figures S2: Gating strategy and representative scatter plots for analysis of maturation in BMDCs (cf. Figure 2a-2b)
3. Figure S3: Gating strategy and representative scatter plots for antigen presentation in BMDCs (cf. Figure 2c)
